# In vitro cytotoxicity and osteogenic potential of quaternary Mg-2Zn-1Ca/X-Mn alloys for craniofacial reconstruction

**DOI:** 10.1038/s41598-022-12490-0

**Published:** 2022-05-18

**Authors:** Somasundaram Prasadh, Manoj Gupta, Raymond Wong

**Affiliations:** 1 Faculty of Dentistry, National University of Singapore, 9 Lower Kent Ridge Road, Singapore, 119083 Singapore; 2 Department of Mechanical Engineering, National University of Singapore, 9 Engineering Drive 1, Singapore, 117576 Singapore

**Keywords:** Cell biology, Molecular biology, Health care, Medical research, Materials science

## Abstract

Cytotoxicity of any biomedical material needs to be investigated for successful application within the human tissues. In this study, manganese in low amounts of 0.3, 0.5 and 0.7 (wt.%) was added to Mg2Zn1Ca alloy using Disintegrated Melt Deposition (DMD) followed by hot extrusion and the extruded alloys were tested for in vitro cytocompatibility using cell viability assays (CCK-8, LDH enzyme release assay, cell cytoskeleton and cell morphology) and in vitro osteogenic potential was evaluated using ALP, Alizarin Red and RT-PCR assays. Addition of manganese improved the cell viability and osteogenic potential in variable concentrations. The Mg2Zn1Ca /0.3 Mn and Mg2Zn1Ca /0.5 Mn alloys showed increased cell viability percentage compared to Mg2Zn1Ca alloys. The cytotoxicity percentage at the end of 24 h culture for Mg2Zn1Ca /0.3 Mn alloys showed lesser cytotoxicity percentage (~ 8%) when compared to the Mg2Zn1Ca /0.5 Mn (~ 13%) and Mg2Zn1Ca /0.7 Mn (~ 16%) samples. All the alloys showed good initial cell attachment, osteogenic potential and cell spreading. The results of this study validates great potential of Mg2Zn1Ca alloys with manganese addition and exhibited great potential for to be used as temporary implant materials in craniofacial reconstruction.

## Introduction

Bone loss caused by congenital defects, tumour resection irradiation, and trauma are common in craniofacial regions^1^. The bone loss occurring due to these conditions must be regenerated or replaced for the critical function of mastication, deglutition and most importantly to maintain the functional harmony and aesthetics of the facial complex^2–4^. Autografts have been a gold standard treatment options for bone loss^5^. Limited donor tissues, tissue morbidity and infections of the affected sites, however, limit the use of autografts^1,6,7^. The cost expense, graft rejection, and the psychological trauma imposed by the second stage surgery will also limit the use of autografts^6,8^. To overcome these limitations, alloplastic materials like Stainless Steel(316L), Cobalt- Chromium(Co-Cr), Titanium Alloys are currently used to fix these autografts or to replace entirely these autografts in combination with other methods like tissue engineered scaffolds^9–11^. The non-resorbable nature of these materials, possibility of infection and need for second stage surgery to remove them can be a major setback in their use for craniofacial reconstructions^12,13^. Therefore use of biodegradable fixation materials would be an ideal option among which magnesium and its alloys have gained increased attention in craniofacial bone tissue engineering in recent times^1,14,15^.

Magnesium and its alloys exhibit superior strength, ductility and degradability compared to its biodegradable counterparts, Zinc and Iron^16^. The elastic modulus (∼ 45 GPa) of magnesium is close to that of human bone (∼ 10–20 GPa) and this helps to avoid stress shielding effects compared to other metallic biomaterials^17,18^. The magnesium in plasma exists as Mg^2^^+^ in concentration of 1.2–1.4 mM and is excreted in urine^19,20^. Increased local degradation of Mg (and its alloys) causes increased hydrogen gas release from implanted site leading to an increase in pH^19^. This increase in hydrogen gas evolution and pH causes localised cell death and cytotoxic reactions which further leads to decreased bone formations^21–23^.

To overcome these challenges, alloying of magnesium with other elements was attempted to lower the degradation rate to reduce the hydrogen gas evolution and in order to increase the cell proliferation and attachment^24,25^. Zinc (Zn) and Calcium (Ca) are the two most common elements alloyed with magnesium. Zinc helps in remodelling of the bone whereas Calcium helps in bone growth and fracture healing^26,27^. However, addition of excess amounts of Zn and Ca leads to the formation of secondary and tertiary phases within the alloy microstructure which increases the galvanic degradations of the MgZnCa alloys and leads to cell death and cytotoxicity ^28^. A cell cytotoxicity study on ternary Mg-Zn-Ca alloys showed that increases in the concentration of Zn and Ca lead to cytotoxicity effects on the mouse pre osteoblast cell lines (MC3T3- E1) and inhibited other essential mineral absorption^25^. To overcome these drawbacks, manganese (Mn) was added to Mg2Zn1Ca alloy, as it had been previously shown to reduce cytotoxicity whilst improving cell proliferation and osteogenic bone regeneration^29,30^.

Manganese (Mn) is an essential trace element for physiological processes, and it is a necessary element for the immune system and a variety of enzymes^31,32^. Mn addition reduces the grain size thereby enhancing grain refinement^33,34^. Addition of Mn forms alpha Mn precipitates which hinders the growth of grains during the process of extrusion. Mn has also been proven to be a promising element in reducing the corrosion rate of magnesium alloys^35,36^. Addition of 0.5 wt. % of Mn reduced the corrosion rate of Mg-2Zn-2Ca alloys ^37^. Mn forms oxide films on the corroding surface and act as a protective barrier preventing the penetration of chloride ions ^38^. Gu et al.^39^ added 1 wt. % of Mn to pure Mg and found that the Mn addition reduced the corrosion rate and increased the mechanical strength of pure Mg^39^. Manganese had been reported to increase the interaction between MC3T3-E1 cells and extracellular matrix by altering integrin activity. This action causes increased cell proliferation, cell spreading and cell viability. Hong et al.^40^ reported increased cell viability and increased cell proliferation of MC3T3-E1 cells cultured with 3D printed Fe–Mn–Ca and Fe–Mn alloys ^40^. Mandal et al.^41^ reported the addition of Mn to porous Fe–Cu alloys increased the initial cell attachment and no cytotoxicity of the adhered cells ^41^. Studies have reported that Mn upregulates the osteogenic gene expression and increases the deposition of collagen fibres ^42–46^. In order to retain the strength enhancements and improve the corrosion resistance, alloying of Mg-Zn-Ca alloy system with a suitable quaternary element can be considered.

The results of literature search indicate that no quaternary alloy with Mg2Zn1Ca with manganese trace addition as investigated in the current study was investigated for in vitro cytotoxicity and osteogenic potential targeting craniofacial applications. Accordingly, in the present study we alloyed manganese (X- 0.3, 0.5 and 0.7%) to Mg2Zn1Ca alloy using Disintegrated Melt Deposition (DMD) followed by hot extrusion and evaluated them for in vitro cytocompatibility and osteogenic potential. DMD is a unique technique which brings together the cost-effectiveness associated with the conventional casting process and the scientific innovativeness and technological potential associated with the spray forming process. However, unlike spray forming, DMD technique uses higher superheat temperatures and lower impinging gas jet velocity. Hot extrusion was used as the secondary processing technology.

The primary focus of the study was to correlate the effect of the addition of manganese on the cytocompatibility properties and osteogenic potential of Mg2Zn1Ca alloys. The in vitro degradation behavior of this alloy along with the post corrosion mechanical strength and microstructural characterization has been reported previously ^47^. The hypothesis for this study was that the developed magnesium alloy with manganese addition will enhance cell proliferation, matrix production, mineral deposition, and osteogenic gene expression of pre-osteoblastic cells compared to the Mg2Zn1Ca alloy.

## Results

### Cytocompatibility

**Figure 1:**
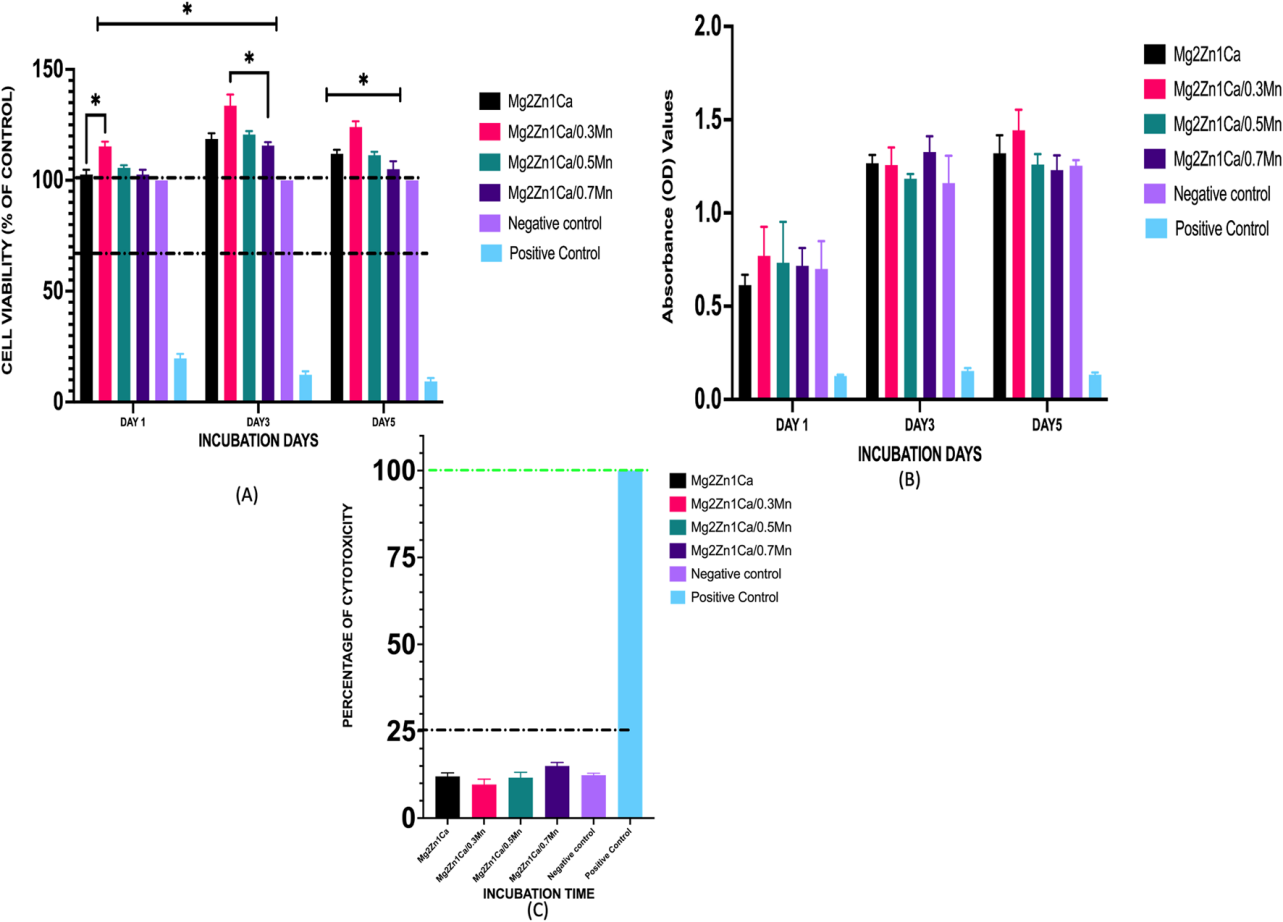
Cell viability of MC3T3-E1 cells cultured with 100% extracts for days 1, 3 and 5 days. (**A**) Cell viability of MC3T3-E1 cells (**B**) Cell Proliferation—Absorbance OD values (**C**) Cytotoxicity Percentage LDH enzyme release. Error bars indicate mean ± standard deviation (*n* = 3, independent samples). Media with cells were used as negative control and 10% dimethyl sulfoxide (DMSO) as positive control. The black dashed line indicates the cut-off between non-toxic and toxic responses (70%) according to ISO 10,993–5 (*P < 0.05) (0.027).

The survival and growth of MC3T3-E1 mouse pre osteoblast cells cultured indirectly with the alloy extracts were quantitatively measured. CCK8 assay was used to evaluate the cell viability and the cell proliferation. The cell viability and cell proliferation were measured at the end of day 1, day 3 and day 5 incubation time point. Figure 1a shows the cell viability percentage of the alloy samples. At the end of day 1 culture, the viability percentage of all the samples were almost equal (~ 100%) with 0.3 Mn samples were significantly on the higher side (~ 110%). There was an increase in cell proliferation on the end of day 3 culture for all the samples. However, there was statistically significant increase in the cell viability percentage for the 0.3 Mn samples (~ 132%). After the end of day 5 culture there was slight decrease in the cell viability percentage for all the samples, with samples having viability percentage more than 100%. The samples showed a high cell viability which is well above the ISO standard for cytotoxicity of materials (70%)^48,49^ . The absorbance OD values shows increased cell proliferation for Mn containing alloys (Fig. 1b). The reduction in the viability percentage on day 5 is attributed to the contact inhibition of cell confluency. The cytotoxicity percentage of the sample were evaluated by the level of LDH enzyme release assay. At the end of 24 h culture, the 0.3 Mn samples showed less cytotoxicity percentage (~ 8%) compared to the 0.5 Mn (~ 13%) and 0.7 Mn (~ 16%) samples. All the alloy compositions were well below the level 0 cytotoxicity percentage (less than 25%)^48,49^ with 0.3 Mn showing the least cytotoxicity (Fig. 1c). There was no statistical difference in the cytotoxicity percentage for all the alloy groups.

### Cell morphology and cytoskeleton organization

**Figure 2:**
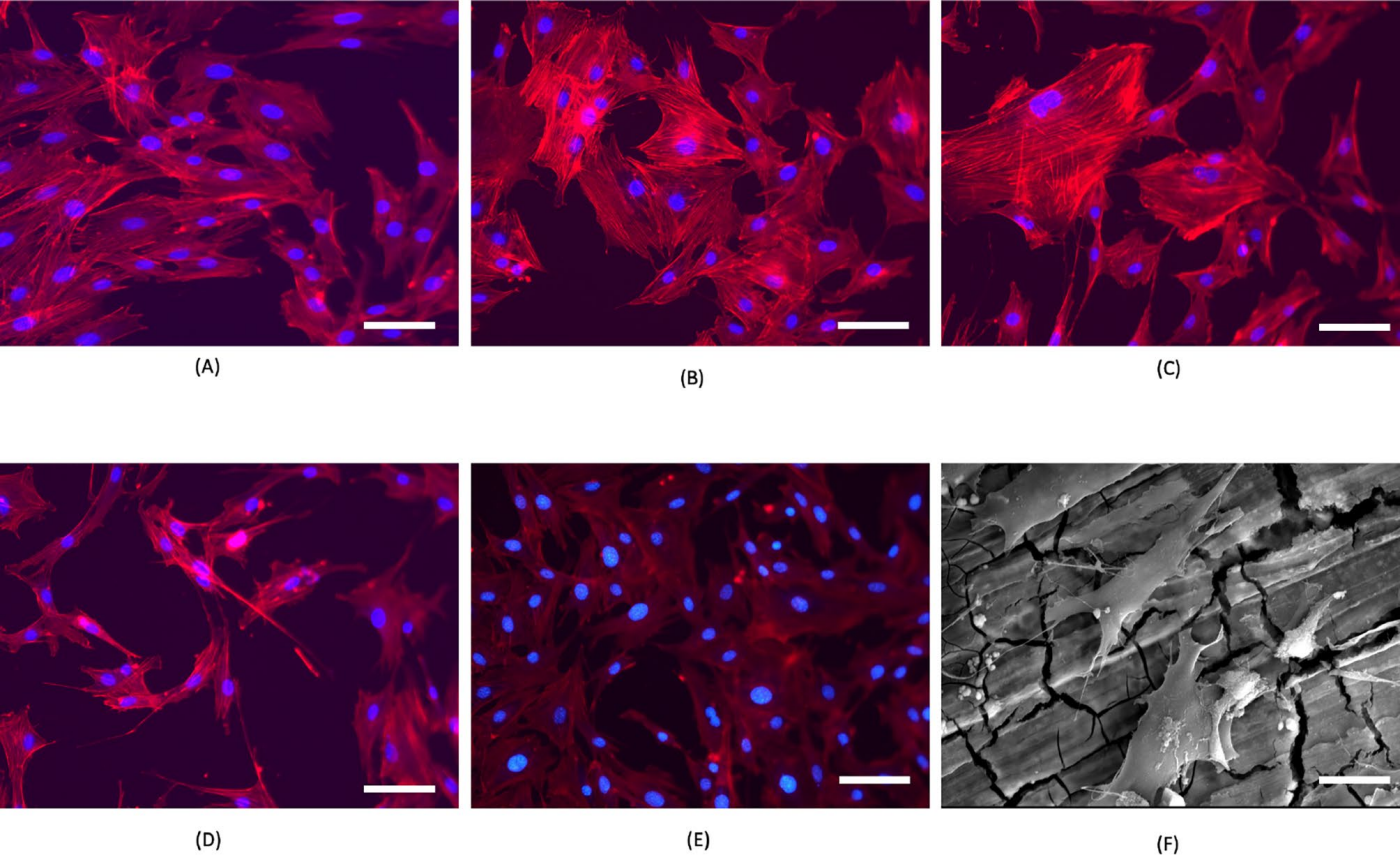
Cytoskeleton staining of MC3T3-E1 cells after incubation for 24 h in different alloy extracts with DAPI for nuclei (blue) and rhodamine phalloidin for F-actin stress fibres (red). Fluorescent microscope images in 20 X magnification and the scale bar indicate 50 µm. (**A**) Mg2Zn1Ca, (**B**) Mg2Zn1Ca/0.3 Mn, (**C**) Mg2Zn1Ca/0.5 Mn, (**D**) Mg2Zn1Ca/0.7 Mn, (**E**) Negative control (Media with cells were used as negative) (**F**) Morphology of MC3T3-E1 cells after incubation for 24 h. Scanning Electron microscope images. (Scale bar indicate 20 µm).

To further understand the direct contact surface interaction between MC3T3 -E1 cells and the experimental sample surface, we examined the cell morphology of MC3T3 -E1 cells after exposure to the extracts and the alloy discs. The cell morphology plays a vital role in the cell viability, cell growth and function. The effect of the composition and elements of the extracts of the material influences the cell morphology and cytoskeleton. After 24 h. culture of the MC3T3 -E1 cells, F actin filaments were extended and elongated in shape for all the experimental groups. The results showed all the alloys were suitable for early cell adhesion. Figure 2 exhibits the morphology of the MC3T3-E1 cells after immersion for 24 h in different extracts. Compared to the Mg2Zn1Ca, the attached cells for the 0.3 Mn and 0.5 Mn alloys extracts show more spreading and superior filopodia extension. The MC3T3-E1 cells cultured in the 0.3 Mn and 0.5 Mn extracts showed good cell adhesion via well-organized F-actin stress fibres (red filaments) compared to 0.7 Mn.

As the Mn concentration increases, aggregation of MC3T3-E1 cells diminishes and the cell status deteriorates slightly, indicate that the 0.3 Mn and 0.5 Mn extracts are favourable to initial attachment and spreading of MC3T3-E1 cells. As can be seen in the Fig. 2, the osteoblasts showed a typical morphology with elongated shape and stress fibres and were arranged in well-defined parallel bundles along the cellular axis.

**Figure 3:**
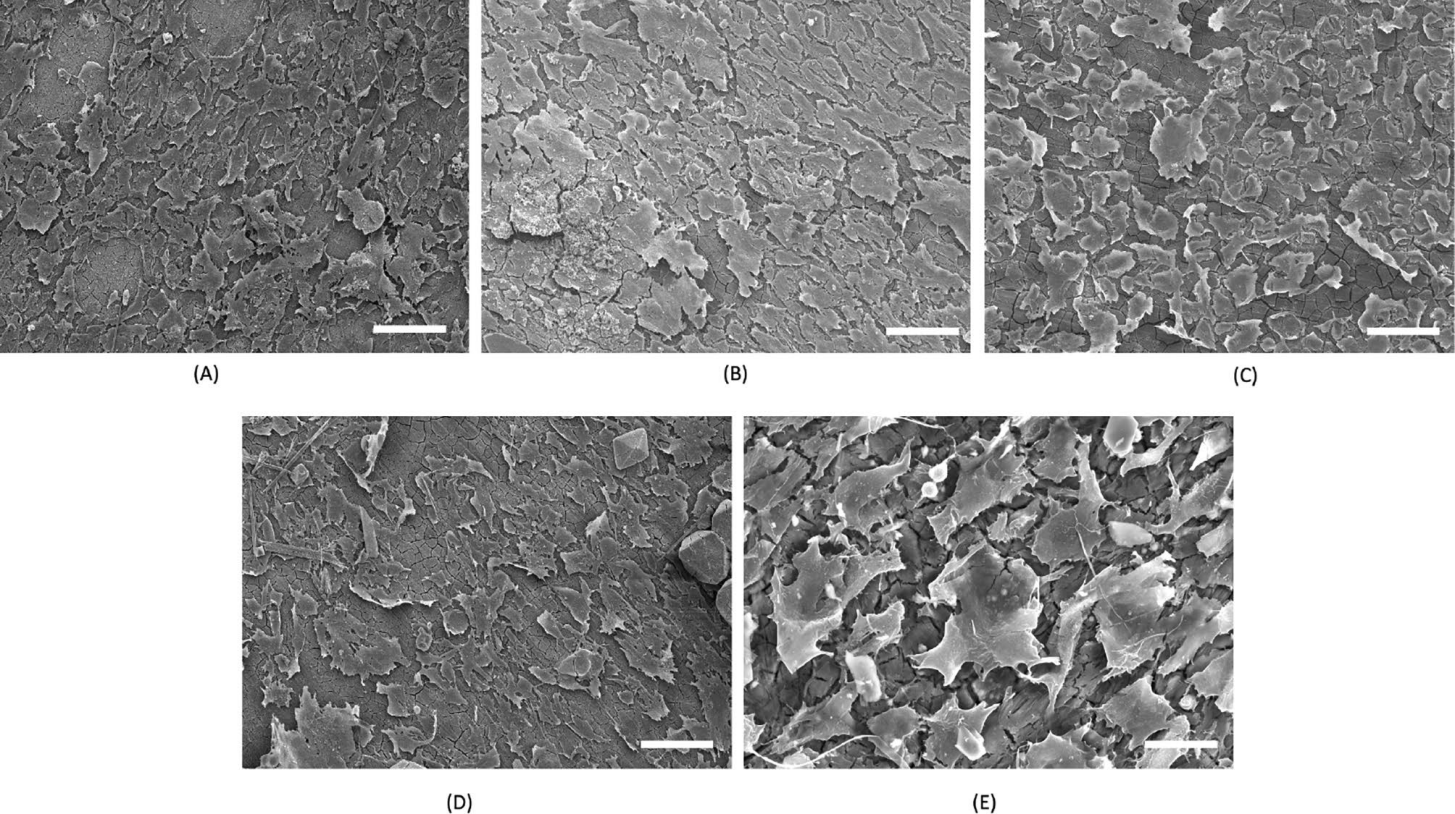
Cell cytoskeleton and morphology—Day 5 culture. After 5 days of direct culture, cells on all the discs were observed to be more flattened and well spread. Images of attachment of MC3T3-E1 cells on selected sample surfaces at 5 Days incubation. (**A**) Mg2Zn1Ca, (**B**) Mg2Zn1Ca/0.3 Mn, (**C**) Mg2Zn1Ca/0.5 Mn, (**D**) Mg2Zn1Ca/0.7 Mn, (**E**) Cytoskeleton cell morphology of MC3T3-E1after day 5. Scale bar 400 μm.

The morphology of MC3T3-E1 cells were further observed using scanning electron microscopy after incubation for 5 days. At the end day 5 culture, there were more spread of cells on the surface of all the alloy discs. The cells showed polygonal morphology and reached almost 100% confluency at the end of day 5 culture. The images indicate that there is good cell attachment and cell viability for all the alloy compositions (Fig. 3).

### Live/dead cell Staining

Figure S4 shows the live and dead cell staining images of MC3T3-E1 cells cultured for incubation days 1, 3 and 5 with the different alloy extracts. The live and dead cell staining was done to observe the cell viability and cell proliferation. At the end of day 1 and day 3 staining showed increased cell proliferation and more live cells (green) with very few dead cells (red) at the end of day 1 and day 3 for all the sample extracts were observed. Fluorescent microscope images indicate Live/Dead staining of primary MC3T3-E1 pre-osteoblasts cultured on extracts for day 1, day 3 and day 5, where viable cells are labelled in green, nucleus of live cells stained in blue, dead cells in red and merge images of live and dead cells. The fluorescent images reveal that the extraction media of Mg-alloys did not impair the viability of MC3T3-E1 pre-osteoblasts throughout the entire observation period. Importantly, very few red-labelled dead cells were observed at the studied time points. Moreover, the results of this study showed a progressive increase in the number of viable osteoblasts over the experimental period of 5 days. Day 1, day 3 and day 5 cell cultures showed increased proliferation for all the groups (green), however few dead cells were observed for all the samples at end of day 5 (red) with 0.7 Mn showing more dead cells (red) (Figure S4).

### Intracellular total protein content and Alkaline phosphatase activity assay

**Figure 4:**
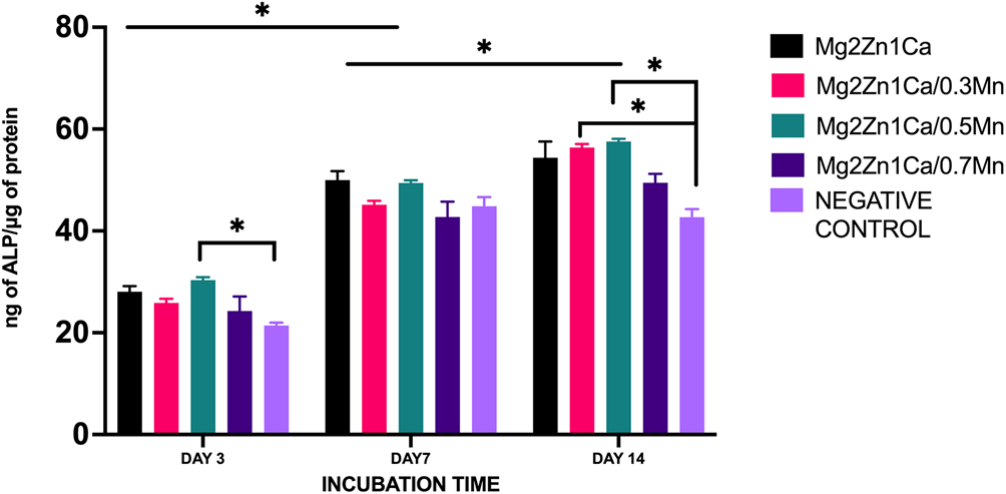
ALP activity of pre osteoblasts osteo induced by different alloys extracts for day 3, day 7 and day 14 culture. Osteogenic medium induced cells culture was used as negative control. The ALP activity of the manganese containing alloy groups shows increased ALP activity compared to Mg2Zn1Ca alloy and the negative control (Osteogenic media with cells). Error bars indicate mean ± standard deviation (*n* = 3, independent samples) (*P < 0.05).

The early stages of osteoblastic differentiation and osteogenesis can be commonly measured by the activity of Alkaline phosphate (ALP). ALP plays a dominant role in the identification of the early osteoblastic differentiation of the MC3T3-E1 cells. The results show that there is an increasing trends in the ALP activity for the incubation time of day 3, day 7 and day 14 (Fig. 4). The cells cultured in the presence of alloy extracts with osteogenic supplements containing manganese showed increased ALP activity compared to Mg2Zn1Ca alloys and the negative control (cells cultured in the osteogenic medium). The order from high to low of the ALP activity level was: "Mg-2Zn-1Ca/0.5Mn > Mg-2Zn-1Ca/0.3Mn > Mg2Zn1Ca > Mg-2Zn-1Ca/0.7Mn". The highest levels were achieved after day 14 incubation whereas day 3 and day 7 showed only slight difference. The results imply high levels of osteogenic differentiation of MC3T3-E1 cells cultured with extracts of manganese alloy groups.

### Extracellular matrix mineralization

**Figure 5:**
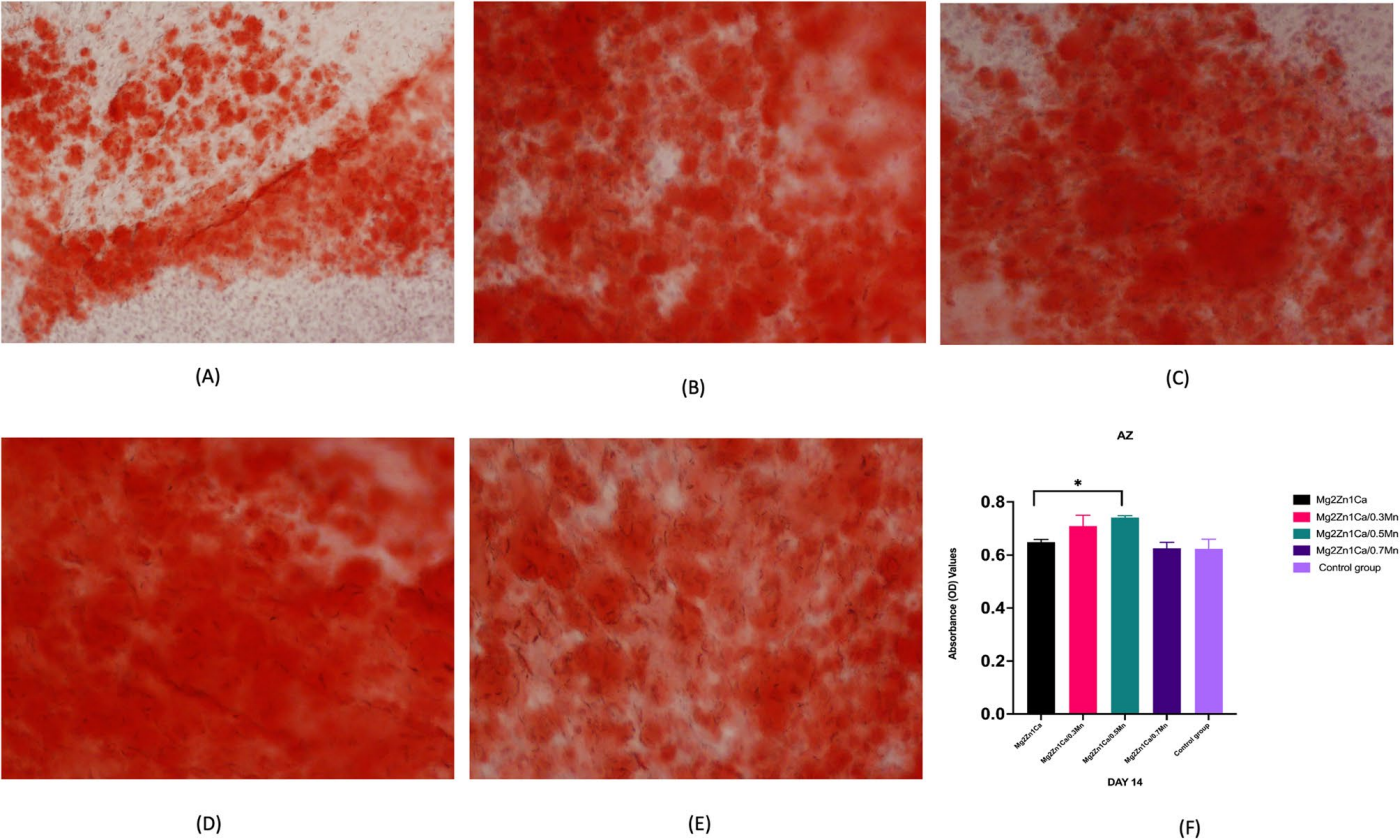
Alizarin red staining was used to determine the extracellular calcium deposition (**A**) Negative Control (Osteogenic media with cells), (**B**) Mg2Zn1Ca, (**C**) Mg-2Zn-1Ca/0.3Mn, (**D**) Mg-2Zn-1Ca/0.5Mn, (**E**) Mg-2Zn-1Ca/0.7Mn, (**F**) Quantitative measurements of Alizarin red activity in MC3T3-E1 cells after 14 day of culture. The cells cultured with osteogenic media without alloy extracts were used as control. Addition of Mn showed increased calcium deposition and staining. Error bars indicate mean ± standard deviation (*n* = 3, independent samples). (*P < 0.05).

Deposition of calcium can be a diagnosis marker for the late stage of osteogenic potential. This can be measured by Alizarin red staining and quantitative analysis. Alloy extracts containing Mn elements showed increase in the calcium matrix deposition compared to control group. 0.3 Mn and 0.5 Mn alloy extracts showed the highest values compared to Mg2Zn1Ca alloys and 0.7 Mn alloys at the end of day 14 cultures. The staining shows extracellular matrix mineralisation and increased visibility. The area stained dark red indicating intense calcification (Fig. 5).

### Osteogenic gene expression

**Figure 6:**
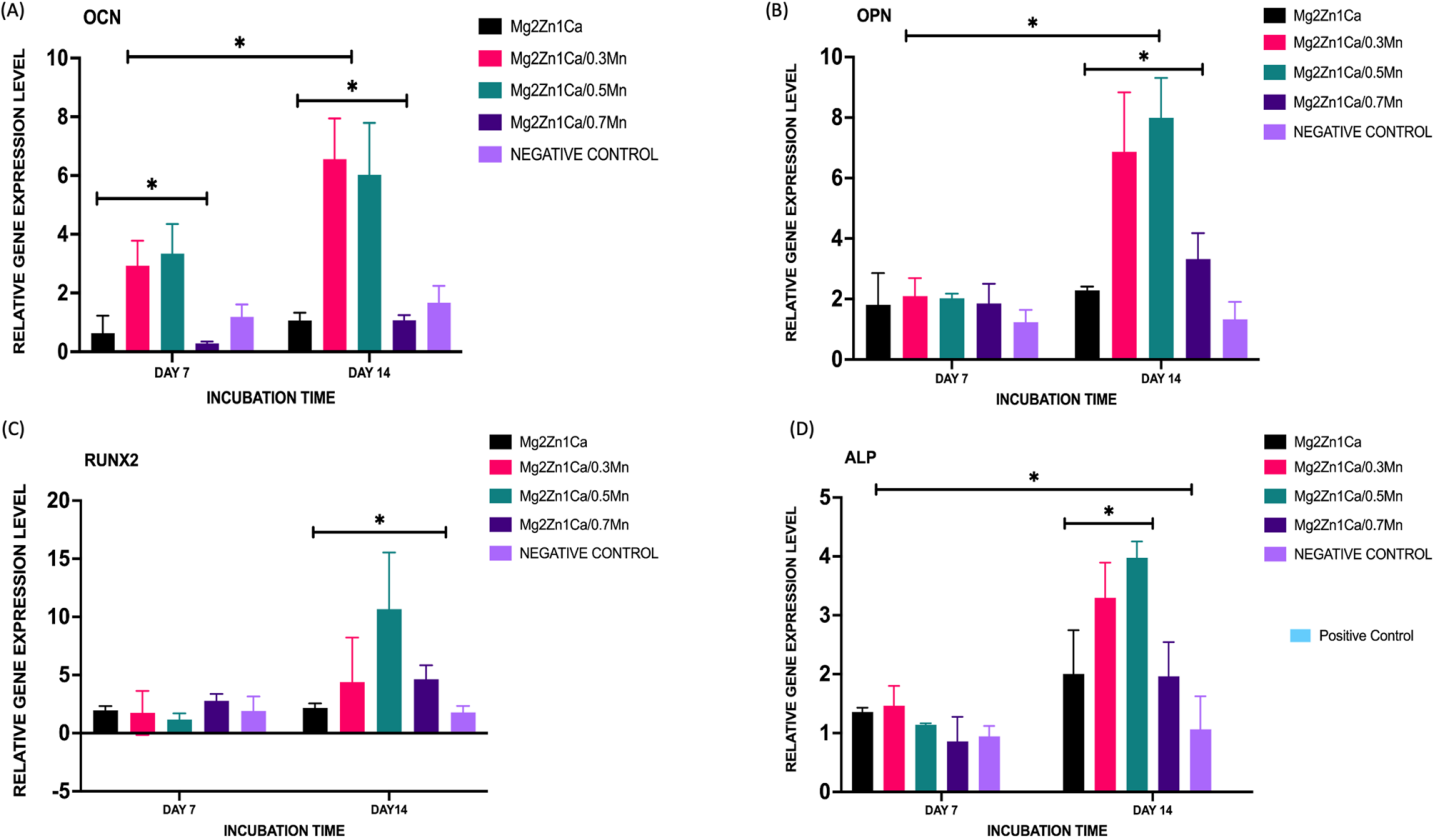
Relative expression level of genes (**A**) Osteocalcin OCN, (**B**) Oteopontin OPN, (**C**) RUNX2, (**D**) ALP in osteoblasts cultured with alloy extracts for 7 and 14 days using SYBR Green RT-PCR. O**s**teogenic Media with cells were used as negative control and 10% DMSO was used as positive control. Error bars indicate mean ± standard deviation (*n* = 3, independent samples). (*P < 0.05) (0.020).

The gene expression level of osteogenesis-related genes for the alloy extracts cultured with MC3T3-E1 cells for day 7 and day 14 are shown in Fig. 6. After day 7 and 14 days of incubation, the expression level of OCN, OPN, RUNX2, ALP was significantly higher than the control group. The results shows that the expression level of genes were upregulated for day 7 and day 14 cultures.

## Discussion

There is a paradigm shift in the biomaterial design to develop a biodegradable metallic biomaterial with less cytotoxicity and more bone regenerating capabilities. Particularly metals containing nutrient elements like Mg, Zn, Ca and Mn in human body plays a promising role in biomedical implant application. In this study we investigated the addition of Mn to the Mg2Zn1Ca alloy and their effect on cytocompatibility and osteogenic potential on MC3T3-E1 pre osteoblast cell lines. The obtained data shows that the addition of Mn increased the cell viability, cell attachment, proliferation and induced in vitro extracellular matrix mineralisation. The results supports the hypothesis that the Mn addition increased the cell differentiation, matrix production, mineral deposition and osteogenic gene expression of pre osteoblast cells.

Biosafety of a potential orthopaedic or craniomaxillofacial material is studied by biocompatibility testing ^50,51^. The cytotoxicity and cell proliferation studies are the most crucial and widely accepted protocol for a cytocompatibility test owing to its rapidness, sensitivity, and simplicity ^39^. In the present study, the results of in vitro cytotoxicity and cell proliferation indicated a favourable performance of the magnesium alloys containing different compositions of Mn (0.3,0.5 and 0.7 Mn). We found that the addition of manganese element to Mg2Zn1Ca alloy did not alter the cytocompatibility of the alloy. With the addition of 0.3 wt. % and 0.5 wt. % Mn, MC3T3-E1 cells showed significant increase in the cell proliferation and cell viability. However, increasing the concentration to 0.7 wt.% of Mn showed decrease in the proliferation rate and viability compared to 0.3 Mn and 0.5 Mn, however, the viability percentage was well above 70% (ISO 10993-5: 2009) (Fig. 1). Among the samples tested, cell viability data showed that 0.3 and 0.5 wt. % Mn exhibited the lowest cytotoxicity and increased cell viability percentage. LDH enzyme release assay results showed addition of manganese for 24 h culture supports the cytocompatibility (Fig. 1). No evident toxic responses were observed in this study according to ISO 10993-5: 2009 the alloy compositions shows level 0 cytotoxicity percentage (less than 25%)^48,52^. Cells in response to the 0.7% Mn containing alloys showed decreased cell proliferation and increased cytotoxicity. This can be attributed to the difference in the sensitivity of the cells towards the particles with increasing Mn concentrations. Similar observations regarding differential sensitivity towards the varying composition of reinforcements added to the magnesium have been made by Lanone et al.^53^ with human alveolar and macrophage cell lines. Similar results were obtained in study done by Wang et al. ^54,55^ which showed the addition of Mn doped Mg_69_Zn_27_Ca_4_ alloys increased the cell viability and showed less cytotoxicity compared to pure Mg ^54,55^. He et al.^56^ investigated the in vitro cytotoxicity of Mg6ZnMn alloys on Human umbilical vein endothelial cells and reported that the addition of Mn did not show any deleterious effect on the cells and there was increased cell proliferation^56^.

Initial cell attachment is the major factor in cell material interactions ensuring a favorable cellular and tissue response^57^. The culture setting and material surface influences the cell adhesion and cell viability. Mn, Mg, Zn, and Ca ions enhance the cell attachment and provide a beneficiary initial stage bone formation and enhance the binding of cell surface receptors and ligand proteins ^58,59^. This can be seen in the cell adhesion results obtained by SEM (Fig. 3). The released Ca, Zn, Mg elements further contributes to the enhanced cytocompatibility. Literature studies on the cytocompatibility of Mg alloys had shown that Zn, Ca and Mn increased cell adhesion and cell proliferation^60,61^. Therefore, the addition of Mn enhances cell adhesion and promotes change in morphology for day 5 incubation (Fig. 3). Mn and Mg, Zn, Ca ions enhance the cell attachment and provide a beneficiary initial stage bone formation and enhance the binding of cell surface receptors and ligand proteins ^58,59^.

The proliferation, extracellular matrix maturation and mineralisation are the three important phases which determine the successful bone formation and bone maturation. This could be achieved by the influence of ALP activity and extracellular bone mineralisation^62,63^. ALP is considered as one of the most important crucial marker of early stage osteoblastic differentiation ^64,65^. At an alkaline pH, the ALP enzyme catalyses the hydrolysis of phosphate esters. The ALP levels will be increased in the early stages of osteoblastic differentiation, whereas the later stages is determined by calcium deposition and extracellular matrix mineralisation. Therefore, the ALP and extracellular matrix mineralisation are the key factors in determining the osteogenic potential of any materials used for implant applications^66,67^. Furthermore, there are various other factors which controls the osteoblast differentiation and bone maturation like, growth factors, transcriptional factors and various signalling pathways. In this study we limited our focus towards ALP, extracellular matrix and gene expression. As shown in Figs. 4 and 5 the ALP quantitative data and Alizarin red staining resulted in increase in the ECM mineralisation compared to the control group. These results are consistent with the previous literature studies on bone marrow stromal cell differentiation^68,69^.

Among the various gene expressions playing a crucial role in bone formation, RUNX2 factor plays an important role in early stages of osteoblastic differentiation^70^. Further RUNX2 helps in activation of the other genes like the OCN and ALP. This could be achieved by the activation of the ALP expression by mechanism of binding to the ALP promoter region. ALP acts as an early stage of differentiation whereas the OCN acts as a marker for later stage of osteoblastic differentiation. The products of OCN indicates the onset of extra cellular matrix deposition^70^. This study results shows that the manganese containing alloy extracts can promote osteogenic differentiation. This could be validated by the results showing increased ALP activity, increased extracellular matrix deposition and gene expressions. There are various literature studies supporting the results stating that the OPN is up regulator of proliferation and differentiation of pre osteoblast cells^66,71–74^. OPN are middle stage markers for osteogenic differentiation and associated with onset of extracellular calcium mineralisation. Furthermore, OPN is shown to cause increased osteoblastic activity promotion ^75^. The OPN gene expression of our study results also correlates to the literature studies indicating increased OPN values (Fig. 6)^66,67^. This could be attributed to the fact that the magnesium alloy extracts inhibits osteoclastic activity. However, the underlying mechanism still needs detailed in depth further studies.

In summary, the synthesised Mg2Zn1Ca/XMn alloys satisfies the basic requirements of an ideal implant biomaterial used for craniofacial applications. The synthesised alloys exhibited superior cytocompatibility and biological functions. With increased cell adhesion, cell proliferation and osteogenic potential, the Mn added alloys can be designed for various bulk and porous implants suitable for various bone defect repairs, fixation plates/screws and intra medullary nails for various bone shaft fracture fixations. The properties of Mn addition can promote controlled release of Mg, Zn and Ca in the early stages of implantation. With the limitations of the study, we have focused on the biological aspect of the alloys in this paper. The in vitro corrosion mechanism of the developed Mg2Zn1Ca/X Mn alloys can be read form our previous published paper attached in the supplementary data (Tables S4, S5; Fig. S5).

## Conclusion

Mg2Zn1Ca-0.3 Mn and 0.5 Mn wt. % alloys can support MC3T3-E1 adhesion and proliferation. Mg2Zn1Ca-0.3 Mn and 0.5 Mn wt. % exhibited higher cell viability and cell proliferation with negligible cytotoxicity to MC3T3-E1 cells. Mineralization of ECM and osteogenic differentiation were significantly enhanced when cells were cultured with Mn. Eventually, all of this would lead to enhanced regulation of genes, cell survival/ growth and differentiation, ECM mineralization, and osteogenesis. In summary, the results of the present study validate that magnesium-based alloys with Mn addition can be used in the application of mandibular/craniofacial reconstruction and bone fixation screws and plates.

## Materials and methods

### Processing

#### Primary processing methods

Disintegrated melt deposition (DMD) is a unique cost-saving processing methodology that combines the advantages of conventional casting and spray processing to produce bulk material using lower disintegrating gas jet velocities. Mg-2Zn-1Ca/X Mn (X- 0.3, 0.5 and 0.7 wt. %) were synthesized using the DMD process. The process involves heating the Magnesium (ACROS Organics, USA) with the alloying element ( 2 wt. % Zinc ~ 149 µm, (spherical) with 99.9% purity (Alfa Aesar USA), 1 wt. % Calcium with 99.9% purity (Alfa Aesar USA) and Manganese (X- 0.3, 0.5 and 0.7 wt. %) ~ 100 µm with 99.9% purity (Alfa Aesar USA).

arranged in a unique sandwich form up to a superheated temperature of 750 °C in a graphite crucible. The alloy melt was stirred at 465 rpm for 5 min to facilitate uniform distribution of the elements and circumvent gravity segregation effects between the matrix and alloying elements. The molten melt was bottom poured and disintegrated by two jets of argon gas oriented normal to the melt stream prior to deposition to obtain 40 mm diameter ingots (Fig. S1)^76^.

#### Secondary processing method

The as-cast billet was soaked at 400 °C for 1 h in a constant temperature furnace prior to extrusion at 350 °C at an extrusion ratio of 20.25:1 using a 150-ton hydraulic press. Colloidal graphite was used as the lubricant. Cylindrical rods of 8 mm diameter were obtained. Detailed information can be found in the following reference. The samples randomly taken from the extruded rods were characterized as per ASTM standards. It is to be noted that the results discussed in the forthcoming chapters are all extruded samples unless specified otherwise. Schematic representation of the extrusion die setup is shown in Fig. S2. The chemical composition of alloy were done by ICP- OES (Table S1)^77^.

#### Cell culture

Osteoblast precursor cell line (MC3T3-E1- Mus musculus—Subclone 14—ATCC CRL-2594TM) was used to check the in vitro cytotoxicity. Alpha-minimum essential medium (MEM) with 10% fetal bovine serum (FBS), 100 U/ mL penicillin and 100 μg /mL streptomycin at 37 °C was used to culture MC3T3-E1 cells in a humidified atmosphere of 5% CO2. Alloy extracts were prepared according to ISO 10,993. The alloy disk samples were immersed in Alpha-minimum essential medium (MEM) supplemented with 10% fetal bovine serum (FBS) and 1% Penicillin/Streptomycin, with a surface area-to-extraction medium volume ratio of 1.25 cm^2^/ml. The samples were then incubated in a humidified atmosphere with 95% humidity and 5% CO_2_ at 37 °C for 24 h. The supernatant was collected, and the obtained extracts were refrigerated at 4 °C. Extracts were analysed for the ion concentration and pH (Table S2/ Fig. S3). Cells were seeded in 96-well plates at a density of 5000 cells in each well for 24 h to allow attachment. The culture medium was then replaced by 100 μL of alloy extracts. The culture media without test alloy extracts was used as negative control and culture medium with 10% dimethyl sulfoxide (DMSO) as positive control. After 1, 3, and 5 days’ incubation, the extracts were replaced by fresh culture medium and 10 μL Cell Counting Kit-8 (CCK-8) was added into each well and incubated at 37 °C in a humidified atmosphere of 5% CO2 for 1 h. The absorbance of mediums were measured at 450 nm using a microplate reader. Optical Density (OD) values obtained from the well plate reader were plotted and the percentage of viable cells were calculated^74^.

#### Lactate dehydrogenase enzyme (LDH) activity

The lactate dehydrogenase (LDH) activity was used as an index of the cytotoxicity in the culture media pure cell culture medium with cells was used for maximum LDH release. CytoTox- One Reagent (Promega) was used for LDH assay. The working solution was prepared by adding 11 ml of assay buffer to 1 vial of substrate powder. MC3T3-E1 (8000 cells) were seeded in 96 well plate and incubated for 24 h at 37 °C in 5% CO2. The culture media without test alloy extracts was used as negative control and culture medium with 10% dimethyl sulfoxide (DMSO) as positive control. The detailed protocol can be obtained from our previous studies (*p* < 0.05; *n* = 3)^78^.

#### Cell attachment and live and dead cell staining

SEM was used to evaluate cell attachment to observe the morphology of adherent cells post 5 days of culture for the samples harbouring cultured MC3T3-E1. Phosphate-buffered saline (PBS) was used to wash the samples. Cell fixation was done with 2.5% glutaraldehyde at 4 °C for 2 h. For post-fixation, the samples were treated with 0.1% osmium tetroxide and dehydrated in a series of ethanol washes (25%, 50%, 75%, 95%, and 100%). The dehydrated specimens were sputter-coated with gold and viewed using SEM. Live-dead cell staining was done using propidium iodide (PI) (Dead cells) stock solution, fluorescein diacetate (FDA), and Hoechst (Blue) and examined using an upright fluorescence microscope ((Leica DMRB, Leitz)^74^.

#### Osteogenic differentiation

##### Intracellular total protein and alkaline phosphatase activity assay

The MC3T3-E1 cells were seeded at a density of 2 X 10^4^ cells/ml in 96 well plates with 100ul of cell suspension and cultured in various alloy extracts supplemented with osteogenic components. The cells were cultured for day 3, day 7 and day 14 time period. At the end of each time point, calorimetric assay was used to evaluate the ALP activity using an ALP reagent containing P- nitrophenyl phosphate (P-NPP) as the substrate and the absorbance was measured at 405 nm. Parallelly the intra cellular protein was measured using Bicinchoninic acid (BCA) protein assay kit. The ALP activity was expressed as μmol min^−1^ mg^−1^ protein and was normalised to the total protein content. Each experiment was carried out in triplicate.

##### Extra cellular matrix mineralisation

Alizarin red staining was used to evaluate the extra cellular matrix mineralisation. The cells were cultured for 14 days with different alloy extracts and after the incubation time point the cells were washed with PBS and fixed with 4% formalin for 35 min and then washed with PBS for 2 times with 5 min interval. The cells were stained with 40 mM Alizarin red staining solution (Sigma) for 30 min RT and washed with deionised water until there is no traces of the stain and the cells were viewed under inverted phase contrast microscope. The quantitative analysis of the calcium deposit was done after 14 days culture by dissolving 10% cetylpyridinium chloride in 10 mM sodium phosphate with pH of 7 and the absorbance values were measured at 620 nm^66,74^.

##### Osteogenic gene expression (RT- PCR)

For real-time PCR analysis, the total RNA was extracted from the cultured cells using a Trizol reagent (Invitrogen) according to the manufacturer’s recommended protocol. 2 μg of RNA was converted to cDNA using iScript cDNA synthesis kit (Bio-Rad). The gene expression of the alloy extracts were examined using q-PCR on Real-Time PCR System (Bio-Rad) with SYBR Green (Bio-Rad). The gene expression levels of osteoblastic markers such as OCN, OPN, RUNX2 and ALP, and GAPDH (control) were measured. GAPDH was used as a control (IDT)^79^. Primer sequences are listed in Table S3.

### Statistical analysis

Mean and standard deviation was computed to summarize the outcomes of interest across different group of materials and over time (in days). To test the mean difference of outcome measures across different type of materials and over time (in days), the assumptions for two- way ANOVA were used. As the assumption for constant variance was violated as tested using Bartlett’s test, the weighted least squared regression (Faraway, 2002) and post-hoc contrast test with Bonferroni correction for multiple comparison were carried out. The data were presented as mean ± standard deviation (*n* ≥ 3, independent samples) and a difference of *P < 0.05 was considered significant. Statistical significance was considered at p-value < 0.05. All statistical analysis was conducted using R (R Core Team, 2020) and emmeans (Russell, 2020) package.

## Supplementary Information

Supplementary Information 1.

Supplementary Information 2.
