# Supplementary material for: In vitro cytotoxicity and osteogenic potential of quaternary Mg-2Zn-1Ca/X-Mn alloys for craniofacial reconstruction
Source: Sci Rep. 2022 May 18;12:8259. doi: 10.1038/s41598-022-12490-0 (PMC9117210; doi:10.1038/s41598-022-12490-0)
Supplement: Supplementary file 1 — Supplementary Information 1. [file 41598_2022_12490_MOESM1_ESM.docx]

- Addition of Mn significantly increased the cell adhesion and proliferation.
- Addition of Mn upregulated the osteogenic gene expression.
- Effect of Mn addition increased the extracellular matrix mineralisation.
- Mg2Zn1Ca/0.3Mn wt.% and Mg2Zn1Ca/0.5Mn wt.% showed excellent cytocompatibility and osteogenic potential.
