# Supplementary material for: In vitro cytotoxicity and osteogenic potential of quaternary Mg-2Zn-1Ca/X-Mn alloys for craniofacial reconstruction
Source: Sci Rep. 2022 May 18;12:8259. doi: 10.1038/s41598-022-12490-0 (PMC9117210; doi:10.1038/s41598-022-12490-0)
Supplement: Supplementary file 2 — Supplementary Information 2. [file 41598_2022_12490_MOESM2_ESM.docx]

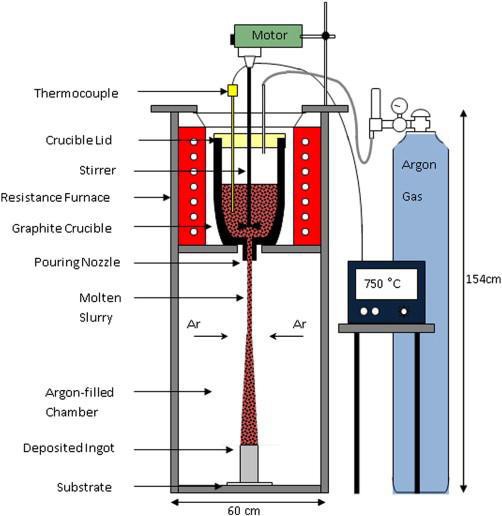


Figure S1: Schematic representation of DMD setup in NUS, Singapore [76]


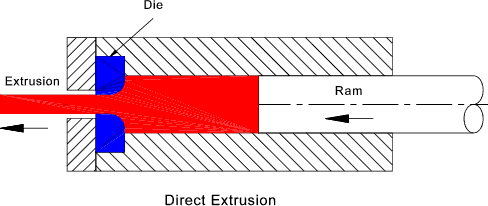


Figure S2: Schematic representation of the hot extrusion process [77]

**
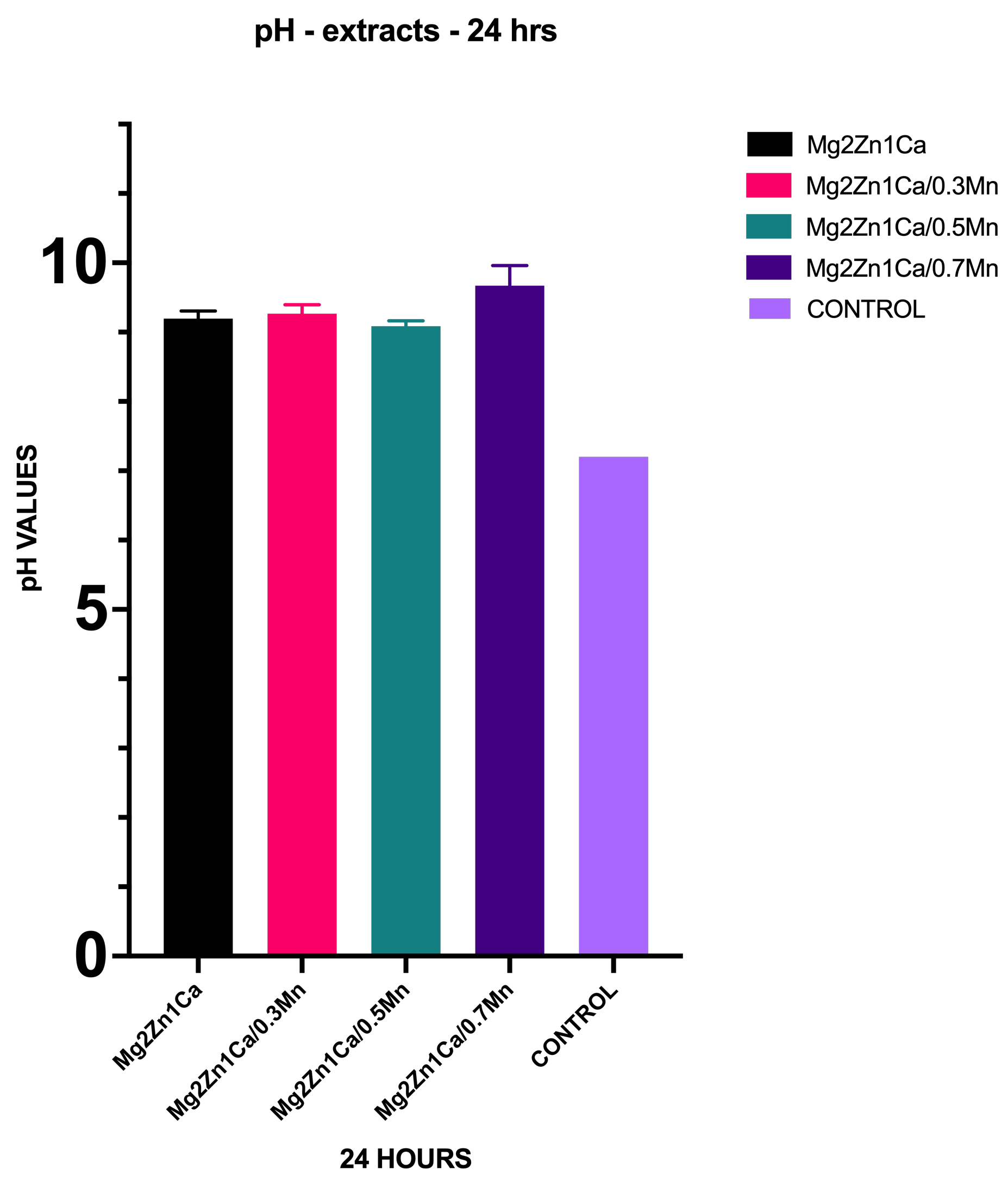
**

Figure S3: pH of alloy extracts in 24 hours


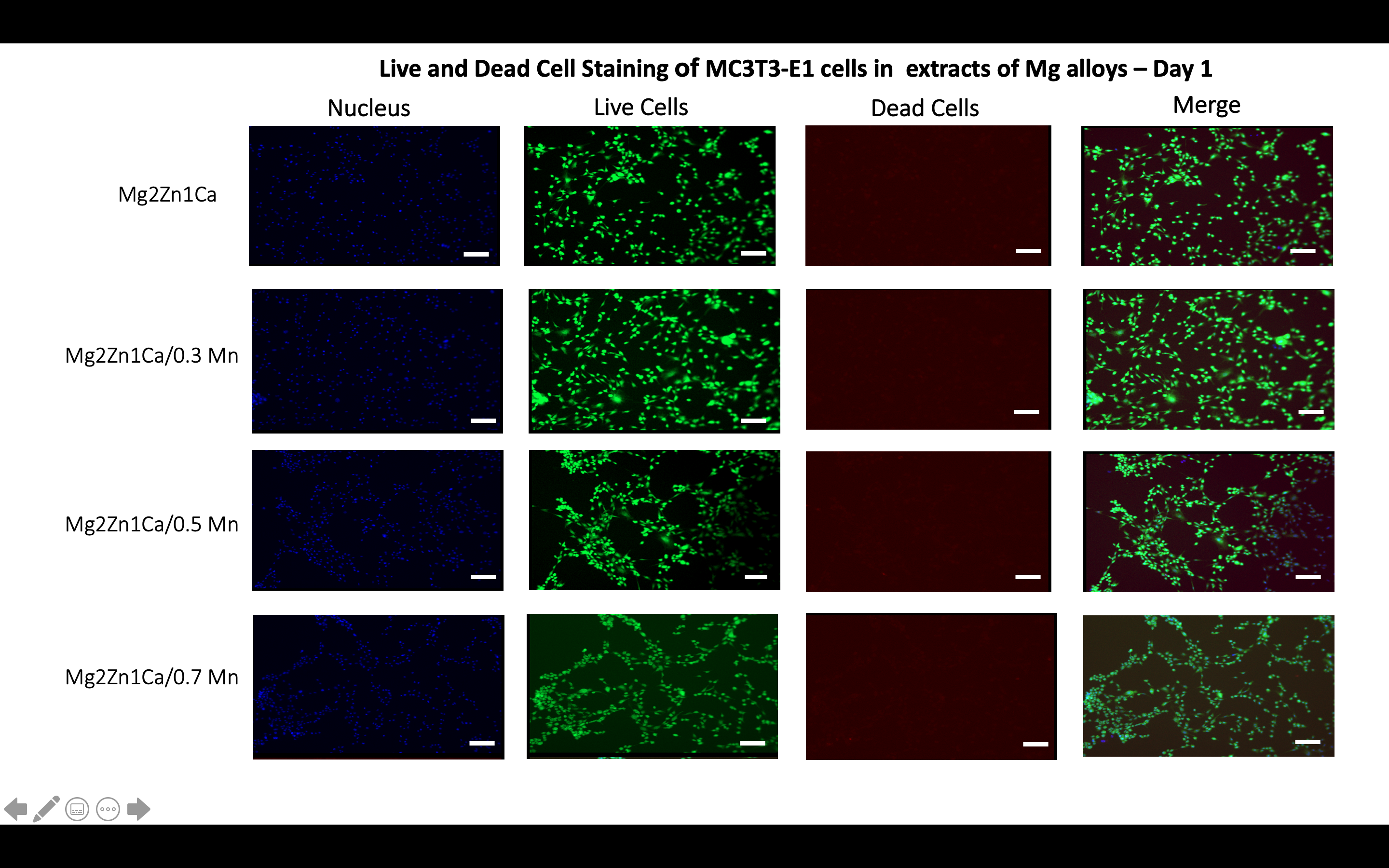


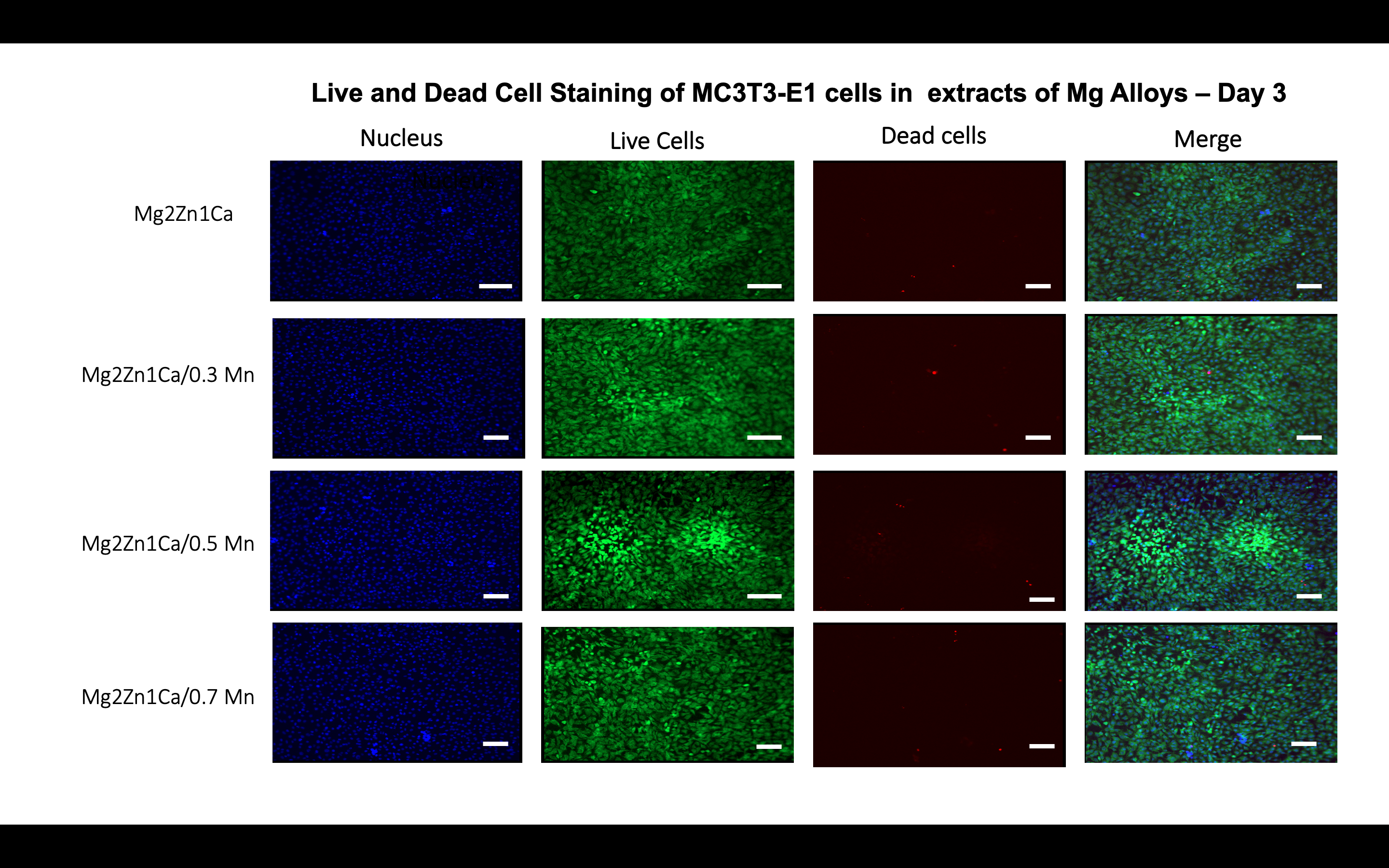


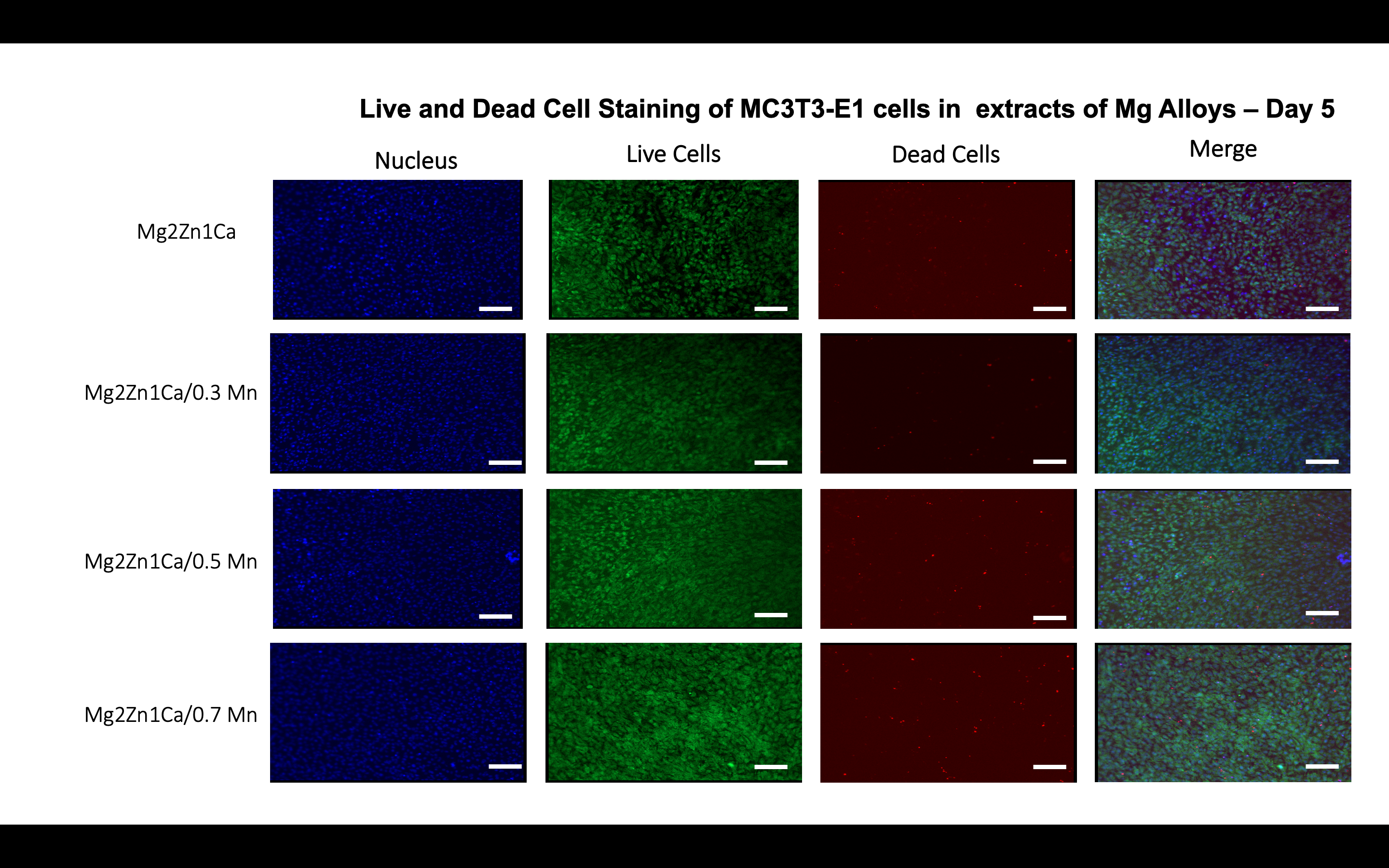


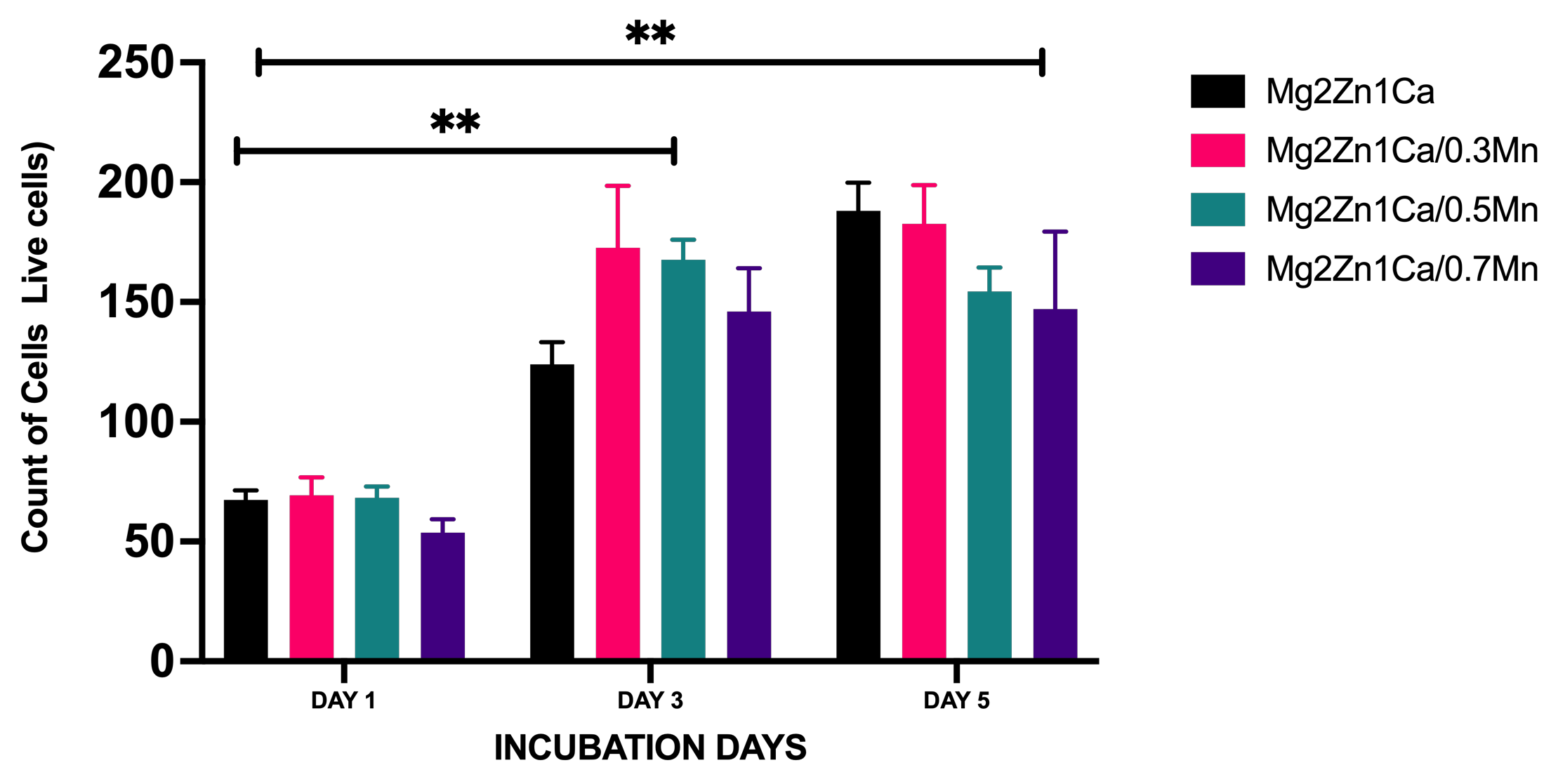

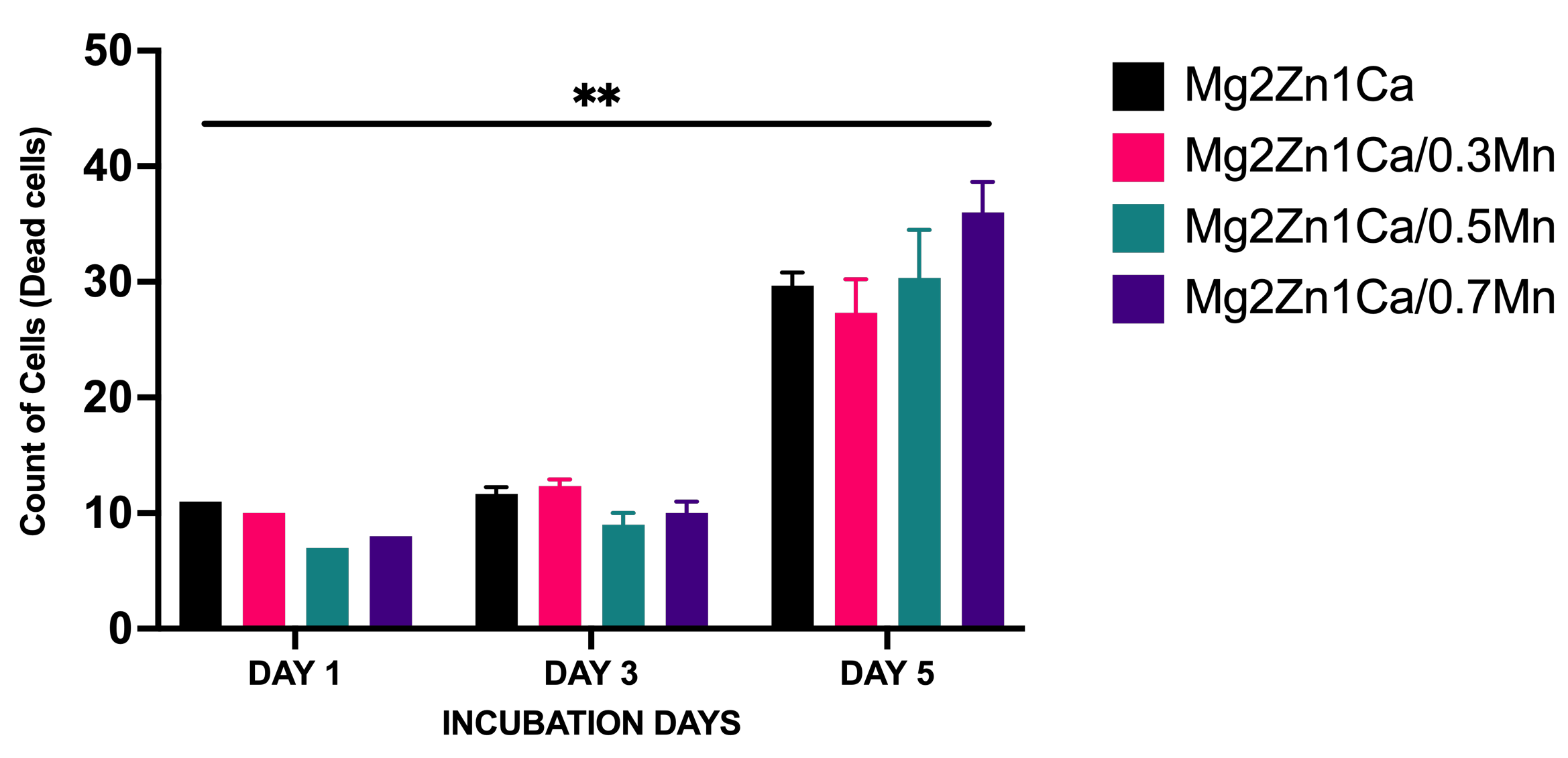


Figure S4 : Fluorescent microscope images in 10 × magnification and the scale bar indicate 200 µm Live/Dead staining of primary MC3T3-E1 pre-osteoblasts cultured on extracts for incubation day 1,3 and 5, where viable cells are labelled in green , nucleus of live cells stained in blue , dead cells in red and merge images of live and dead cells. Bar graphs shows the FITC intensity quantification for the live cells cultured for day 1, 3 and 5 incubation for the alloy extracts. Cells were counted from the images taken from 3 samples of each material using image J software (n = 3, independent images) P<0.0001(**)

Table S1: Chemical composition of alloy (wt. %) according to the ICP-OES.

| Alloys | Mg | Zn | Ca | Mn |
| --- | --- | --- | --- | --- |
| Mg2Zn1Ca | Bal. | 1.89 | 0.93 | 0 |
| Mg2Zn1Ca0.3Mn | Bal. | 1.88 | 0.97 | 0.15 |
| Mg2Zn1Ca0.5Mn | Bal. | 1.98 | 0.94 | 0.49 |
| Mg2Zn1Ca0.7Mn | Bal. | 1.92 | 0.99 | 0.66 |

Mg: Magnesium; Zn: Zinc; Ca: Calcium; Mn: Manganese

Table S2 : Chemical composition of alloy extracts according to the ICP results (ppm).

| Alloys | Mg | Zn | Ca | Mn |
| --- | --- | --- | --- | --- |
| Mg2Zn1Ca | 131 | 0.78 | 58.9 | 0 |
| Mg2Zn1Ca0.3Mn | 190 | 0.88 | 52.8 | 0.04 |
| Mg2Zn1Ca0.5Mn | 161 | 0.99 | 59.9 | 0.11 |
| Mg2Zn1Ca0.7Mn | 168 | 0.91 | 58.6 | 0.14 |

Mg: Magnesium; Zn: Zinc; Ca: Calcium; Mn: Manganese

Table S3 : Primer sequences for real-time PCR analysis.

| **Primers** | **Sequence** | **Notes** |
| --- | --- | --- |
| mRunx2_266bp-F | 5'-cttcattcgcctcacaaac-3' | NM_001146038.2 |
| mRunx2_266bp-R | 5'-gtcactgcgctgaaga-3' | NM_001146038.2 |
| mOCN_149bp-F | 5'-tgaacagactccggcg-3' | NM_007541.3 |
| mOCN_149bp-R | 5'-gataccatagatgcgtttg-3' | NM_007541.3 |
| mAlp_453bp-F | 5'-ggggacatgcagtatgaatt-3' | [NM_007431.3](https://www.ncbi.nlm.nih.gov/entrez/viewer.fcgi?db=nucleotide&id=563317856) |
| mAlp_453bp-R | 5'-ggcctggtagttgttgtgag-3' | [NM_007431.3](https://www.ncbi.nlm.nih.gov/entrez/viewer.fcgi?db=nucleotide&id=563317856) |
| mOPN_139bp-F | 5'-tcaggacaacaacggaaaggg-3' | [NM_001204201.1](https://www.ncbi.nlm.nih.gov/entrez/viewer.fcgi?db=nucleotide&id=323668332) |
| mOPN_139bp-R | 5'-ggaacttgcttgactatcgatcac-3' | [NM_001204201.1](https://www.ncbi.nlm.nih.gov/entrez/viewer.fcgi?db=nucleotide&id=323668332) |
| mGAPDH_223bp-F | 5'-aactttggcattgtggaagg-3' | [NM_008084.3](https://www.ncbi.nlm.nih.gov/entrez/viewer.fcgi?db=nucleotide&id=576080553) |
| mGAPDH_223bp-R | 5'-acacattgggggtaggaaca-3' | [NM_008084.3](https://www.ncbi.nlm.nih.gov/entrez/viewer.fcgi?db=nucleotide&id=576080553) |

**Highlights of the degradation of Mg2Zn1Ca/X Mn alloys [47]**

*This article is an open access article distributed under the terms and conditions of the Creative Commons Attribution (CC BY) license (https:// creativecommons.org/licenses/by/ 4.0/).*

1. Mg–2Zn–1Ca/X Mn (X = 0.3, 0.5, and 0.7) alloys were developed using disintegrated melt deposition followed by hot extrusion.
2. The corrosion resistance of Mg–2Zn–1Ca alloys was improved with the addition of 0.5 wt.% Mn element.
3. The enhanced corrosion resistance is due to improved corrosion product film.
4. Post-corrosion compressive strength of Mg–2Zn–1Ca/0.3Mn and Mg-2Zn1Ca/0.5Mn stabilized after day 21.

#
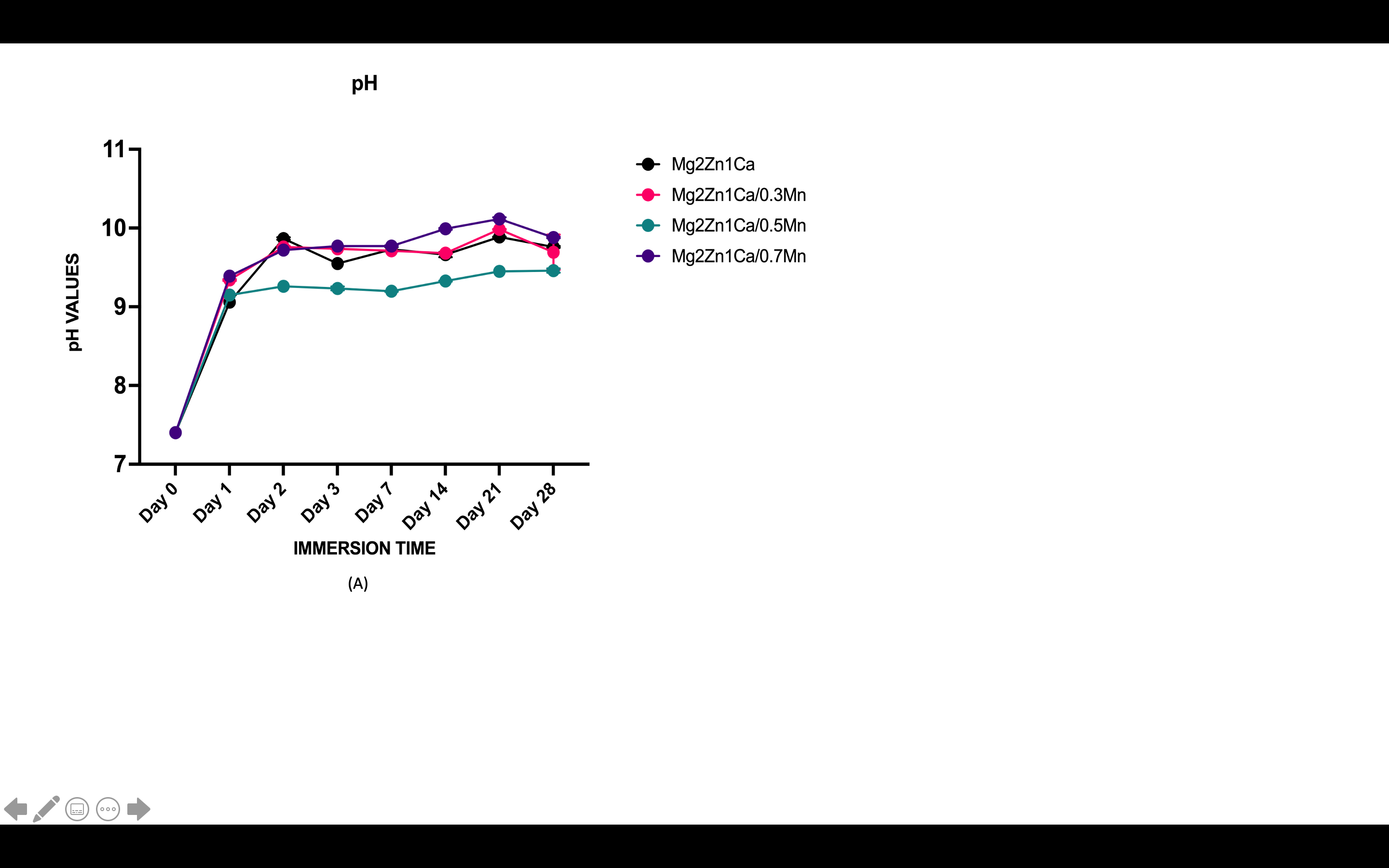

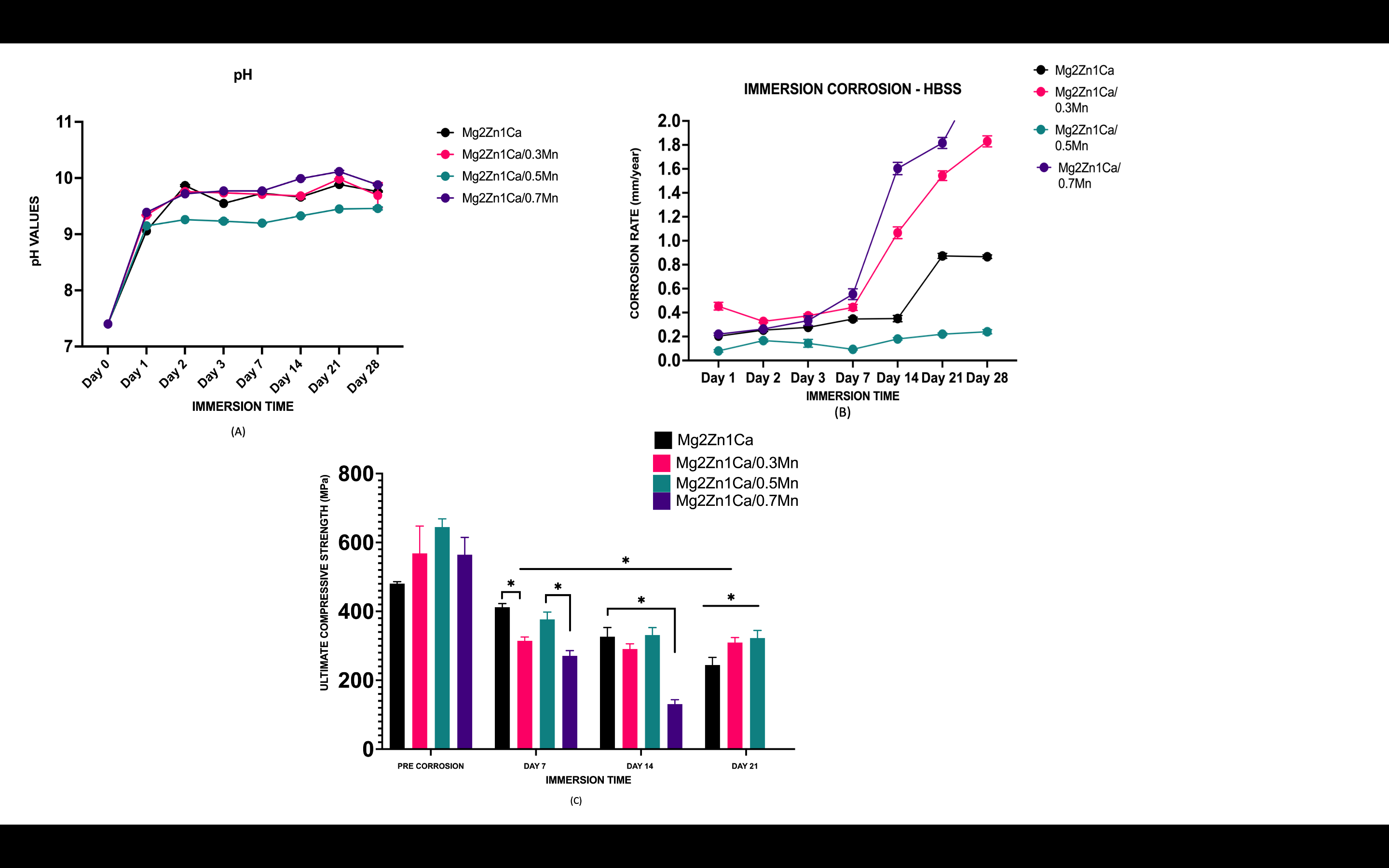

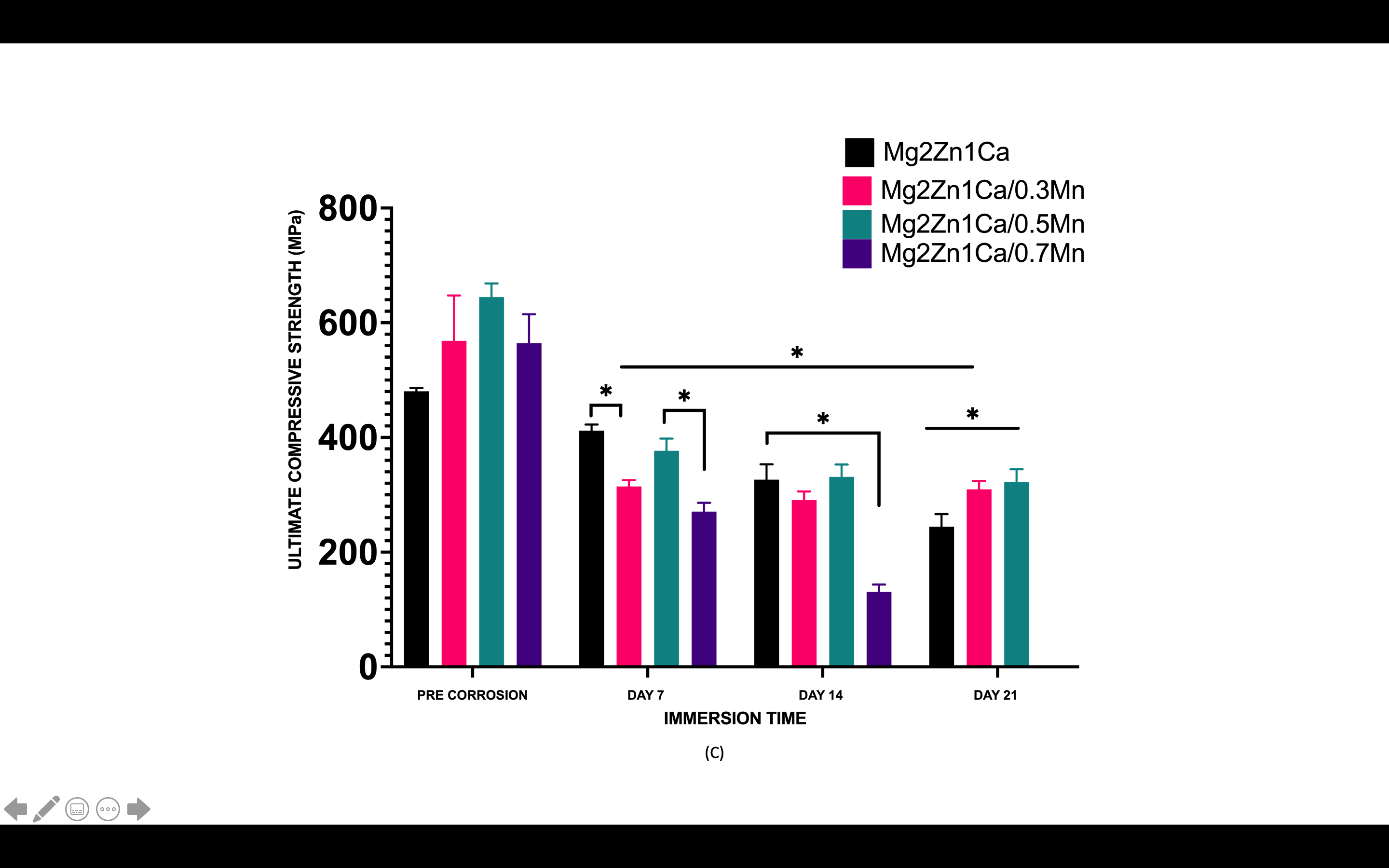


Figure S5: Immersion corrosion rates, pH and post corrosion compressive strength of magnesium alloys in HBSS solution. (A) pH vs Time of immersion; (B) Corrosion rate vs Time of immersion (C) Post corrosion Ultimate Compressive Strength (UCS). The loading direction was parallel to the extrusion axis direction [47].

**Table S4: Corrosion rates of alloys for day 28 immersion in HBSS [47].**

| **Immersion time (Days)** | **Corrosion rate (mm . year^-1^)** | | | |
| --- | --- | --- | --- | --- |
|  | **Mg-2Zn-1Ca** | **Mg-2Zn-1Ca/0.3Mn** | **Mg-2Zn-1Ca/0.5Mn** | **Mg-2Zn-1Ca/0.7Mn** |
| Day 1 | 0.19 | 0.43 | 0.07 | 0.21 |
| Day 2 | 0.26 | 0.32 | 0.16 | 0.26 |
| Day 3 | 0.27 | 0.36 | 0.13 | 0.31 |
| Day 7 | 0.33 | 0.42 | 0.09 | 0.50 |
| Day 14 | 0.32 | 1.10 | 0.19 | 1.56 |
| Day 21 | 0.89 | 1.58 | 0.22 | 1.79 |
| Day 28 | 0.87 | 1.82 | 0.25 | 2.15 |

**Table S5: Comparison of the lowest corrosion rate of the magnesium alloys containing manganese elements using SBF and Hank's solution [47].**

| Alloys | Solution | Corrosion rate (mm/y) |
| --- | --- | --- |
| **Mg-2Zn-1Ca/0.5Mn (This work)** | **Hank’s** | **0.25** |
| Mg-4Zn-0.5Ca-0.75Mn | Hank’s | 0.12 |
| ZK60 | Hank’s | 0.32 |
| MAO-coated ZK60 | Hank’s | 0.003 |
| Mg-2Zn-Ca-0.5Mn | Hank’s | 1.58 |
| Mg-2Zn-Ca-0.5Mn-1.3Ce | Hank’s | 1.34 |
| Mg–2Ca–0.5Mn–2Zn | SBF | 1.78 |
| Mg–2Ca–0.5Mn–4Zn | SBF | 2.21 |
| Mg–2Ca–0.5Mn–7Zn | SBF | 3.98 |
| Mg-Zn-1.5-Ca-1.1Mn | Hank’s | 1.40 |
| 6h-coated Mg-2Zn-Ca-0.5Mn-1.3Ce | Hank’s | 1.29 |
| Mg-Zn-Ca BMGC | SBF | 0.26 |
| Annealed Mg-Zn-Ca | SBF | 1.20 |

*SBF: Simulated Body Fluid
